# Nocturnal Hypoxemia Impacts Right Ventricle Diastolic Function in Obstructive Sleep Apnea: A Retrospective Observational Study

**DOI:** 10.3390/jcm9010162

**Published:** 2020-01-07

**Authors:** Carla Scotti, Roberto Porta, Adriana Olivares, Laura Comini, Angelo Cinelli, Simonetta Scalvini, Michele Vitacca

**Affiliations:** 1 Istituti Clinici Scientifici Maugeri IRCCS, Cardiac Rehabilitation of the Institute of Lumezzane, 25065 Lumezzane, Italy; angelo.cinelli@icsmaugeri.it (A.C.); simonetta.scalvini@icsmaugeri.it (S.S.); 2 Istituti Clinici Scientifici Maugeri IRCCS, Respiratory Rehabilitation of the Institute of Lumezzane, 25065 Lumezzane, Italy; roby.door67@gmail.com (R.P.); michele.vitacca@icsmaugeri.it (M.V.); 3 Istituti Clinici Scientifici Maugeri IRCCS, Scientific Direction of the Institute of Lumezzane, 25065 Lumezzane, Italy; adriana.olivares@icsmaugeri.it (A.O.); laura.comini@icsmaugeri.it (L.C.)

**Keywords:** obstructive sleep apneas (OSAs), right ventricular diastolic dysfunction, nocturnal desaturation, rehabilitation

## Abstract

Obstructive sleep apnea (OSA), although a growing healthcare problem and documented risk factor for cardiovascular diseases, is still under-diagnosed in cardiac patients. To investigate the correlation between OSA and echocardiographic parameters of right ventricle diastolic (RVD) dysfunction, in particular trans-tricuspid E-wave deceleration time (EDT), we retrospectively analyzed data of 103 pure (comorbidity-free) OSA patients with comprehensive echocardiographic examination (ETT). Apnea/hypopnea index (AHI), oxygen desaturation index (ODI), mean nighttime oxyhemoglobin saturation (SpO_2_), time elapsed with SpO_2_ < 90% (T90) and mean peak desaturation of nocturnal events (Mdes, graded as mild, medium or severe) were compared with echocardiographic parameters. We found RVD dysfunction present in 58.3% of patients. Altered EDT correlated significantly with mean SpO_2_, T90, and Mdes (*p* < 0.01, all). Nocturnal desaturators had a significantly worse EDT than non-desaturators (*p* = 0.027) and a higher risk of prolonged EDT (odds ratio, OR = 2.86). EDT differed significantly according to Mdes severity (*p* = 0.005) with a higher risk of prolonged EDT in medium/severe vs. mild Mdes (OR = 3.44). EDT detected the presence of RVD dysfunction in 58.3% of our pure OSA patients. It correlated poorly with AHI severity but strongly with nocturnal desaturation severity, independently of age. This ETT marker may be useful for deciding appropriate diagnostic and therapeutic strategies.

## 1. Introduction

Obstructive sleep apnea (OSA) is a clinical condition characterized by recurrent intermittent episodes of apnea and hypopnea during sleep, generally associated with nocturnal oxyhemoglobin desaturation equal to or greater than 3% of the basal value [1,2,3]. A nocturnal eight-channel cardiorespiratory polygraphy (nCRP) provides objective and measurable parameters to define the OSA pattern, and quantifies the events of apnea/hypopnea (apnea/hypopnea index, AHI) giving information for the diagnosis and severity classification [4,5,6]. OSA is a growing healthcare problem and its increased prevalence is partly attributable to current pandemic obesity. Predisposing factors are described in the literature [7].

The syndrome appears to be associated with an increased risk of cardiovascular mortality and morbidity [7,8,9,10]. Possible complications include pulmonary and systemic hypertension, arrhythmias, coronary artery disease, heart failure, right and left ventricular dysfunction, sudden death, and stroke [9,11]. The presence of OSA is associated with an increased relative risk of developing heart failure (2.38-fold) regardless of other known risk factors [12]. Right heart failure and cor pulmonale are not common in the natural history of isolated OSA in which pulmonary hypertension (PH) is usually mild to moderate [13]. Prevalence of PH of varying degrees in patients with OSA is judged to be close to 17% and implies a poor prognosis [13,14,15,16], depending on the OSA severity. However, the pulmonary artery pressure (PAP) assessment method, time of measurement, and other factors may influence this result [17].

Confirming the key role played by apnea events in the pathogenesis of cardiac dysfunction, an early treatment of OSA with continuous positive airway pressure (CPAP) can lead to an improvement in systolic and diastolic left ventricle (LV) and right ventricle (RV) function [18,19,20,21]. However, sleep respiratory disorders such as OSA are still under-diagnosed, and a clinical–instrumental pathway to screen for the presence of sleep pathology is generally not included in the cardiac patient’s assessment procedure. Conversely, in patients admitted for respiratory rehabilitation with confirmed OSA, the impact of nocturnal hypoxemia on RV relaxation has also as yet not been investigated or it has been investigated in a low number of patients. 

Hence, in this study we focused our attention on a specific marker of RV diastolic (RVD) function: the early trans-tricuspid diastolic inflow curve (or E-wave) deceleration time (EDT), measured by pulsed wave (PW) Doppler. EDT is considered to be one of the most useful indicators of RVD alterations [22,23,24,25]. Our primary aim was to evaluate how many patients admitted to our out-patient sleep service for nCRP, with nocturnal apnea and free from significant comorbidities, i.e., pure OSA patients, showed alterations of RVD function, as evaluated by EDT. The secondary aim was to correlate EDT with nCRP parameters indicative of the severity of OSA. Moreover, other RV function and morphology parameters were evaluated to investigate the association with OSA: RV wall thickness, RV diameter, velocity of the longitudinal movement of the lateral tricuspid annulus in the ventricular ejection phase, and PAP. Influence of minor comorbidities (significant comorbidities were excluded as per the study criteria), such as presence of systemic arterial hypertension, was evaluated to find any impact on RVD function and OSA severity.

## 2. Experimental Section

The protocol was approved by the Ethical Committee of the Istituti Clinici Scientifici (ICS) Maugeri, Pavia, Italy (CEC 2275 12 March 2019). All patients gave informed consent for the scientific use of their data. 

### 2.1. Study Design

This was a retrospective observational cohort study of institutional data. Data from patients with a first diagnosis of OSA, of both sexes and aged ≥18 years, referred to our respiratory sleep disorders service for problems of snoring and daytime sleepiness during the years 2007–2016 were considered. Patients with a first confirmed diagnosis of OSA, obtained by nCRP, without significant cardiac, respiratory or neurological comorbidities, and who underwent a conventional complete transthoracic cardiac echo-color Doppler study (ETT), including tissue Doppler imaging (TDI), were selected. ETT had been performed by the cardiologist, based on the pneumologist’s indication, in order to analyze in depth the relationship between cardiac function and OSA severity in the case of ‘pure’ OSA patients. The ETT was performed before starting treatment with CPAP or non-invasive mechanical ventilation (NIMV) and under conditions of clinical stability.

**Figure 1:**
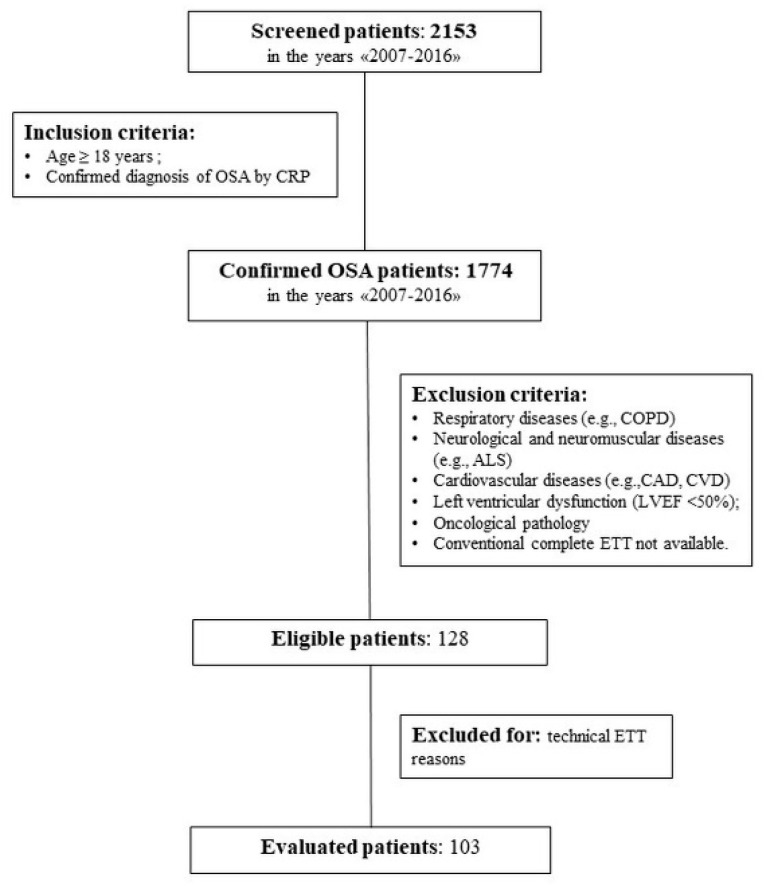
Study flowchart. Legend: nCRP = nocturnal eight-channel cardiorespiratory polygraphy; OSA = obstructive sleep apnea; COPD = chronic obstructive pulmonary disease; ALS = amyotrophic lateral sclerosis; CAD = coronary artery disease; CVD = coronary vascular disease; LVEF = left ventricular ejection fraction; ETT = comprehensive transthoracic two-dimensional and Doppler echocardiography examination.

For further details, see the study flow chart in Figure 1.

### 2.2. Measurements

Data were retrieved from the specialist’s report compiled at the clinical visit and from nCRP and ETT monitoring records stored in our institutional archive. All data included a single time evaluation. Only clinical records with demographic data and complete nCRP and ETT reports were included in the analysis (Figure 1).

#### 2.2.1. Night Cardiorespiratory Monitoring

nCRP evaluations were carried out using the portable eight-channel device EMBLETTA Z10 and EMBLETTA PDS system (Embla Systems, Kanata, Ontario, Canada),in accordance to the consensus statement regarding the use of portable monitors by the Portable Monitoring Task Force of the American Academy of Sleep Medicine (AASM) [4].This nCRP included a combination of sensors, i.e., nasal cannula to detect apneas, hypopneas and snoring, respiratory thoracic and abdominal movements to detect a respiratory effort, pulse oximetry to detect a saturation value and heart rate, pulse transit time, and body position.

Apnea was defined as a complete stopping of airflow lasting more than 10 s. Hypopnea was defined as 30% or more reduction in respiratory airflow lasting more than 10 s and accompanied by a decrease of more than 4% in oxygen saturation. The average number of episodes of apnea and hypopnea per hour of sleep was defined in the apnea/hypopnea index (AHI). The OSA severity was classified according to the AHI in accordance with the AASM 2007 [6]. The scoring and analysis of the tracks were done by an expert technician and a pneumologist dedicated to sleep medicine [4,6,26]. We considered the following parameters:

- AHI: expressed as number of events/h, and OSA severity classified as mild (<15/h), moderate (15–29/h), or severe (≥30/h);
- Oxygen desaturation index (ODI), expressed as number of events/h;
- Mean nighttime oxyhemoglobin saturation (mean SpO_2_:), expressed as a percentage;
- Time elapsed with SpO_2_ < 90% (T90), expressed as a percentage of the total monitoring nocturnal time; two classes of patients were evaluated: non-desaturators (T90 < 30%) and desaturators (T90 ≥ 30%) [27];
- Mean desaturation peak values of nocturnal events (Mdes), expressed as a percentage, and relative subdivision into three severity groups: mild (≥89%), moderate (between 85% and 89%), or severe (≤85%) referred to, respectively, Mdes 89, Mdes 85–89, and Mdes 85;
- Apnea duration: mean duration time of sleep apnea/hypopneas, expressed in seconds.

#### 2.2.2. Comprehensive Transthoracic Two-Dimensional and Doppler Echocardiography Examination

ETT was carried out with a commercial ultrasound system (Vivid 7 and Vivid E9, GE Healthcare, Horton, Norway) and a 2.5 MHz probe. All measurements (M-Mode, 2D, color-Doppler and tissue Doppler imaging) were performed and interpreted according to the recommendations of the European Association of Echocardiography/American Society of Echocardiography [23,24,25,28]. The analysis was carried out by two cardiologists. For the purposes of the study, only some specific ETT parameters obtained during the acquisition and reporting were retrospectively extrapolated:

- EDT: deceleration time of the E-wave of the trans-tricuspid diastolic flow curve, acquired from the apical four-chamber view. This was the RVD function parameter (normal values for diastolic function <240 ms; PW Doppler modality);
- RV diameter: transverse diameter of the right ventricle, recorded in correspondence to the tract of inflow in the telediastolic phase, from apical four-chamber view (2D modality);
- RV wall thickness: parietal end-diastolic thickness of the RV, recorded from parasternal long axis view (M-Mode modality);
- RV-Sm: pulsed-wave TDI was recorded at the lateral tricuspid annulus from four-chamber view, in the ventricular ejection phase (TDI modality);
- Pulmonary arterial systolic pressure (PASP), calculated using the modified Bernoulli equation and conventional Doppler tricuspid regurgitation, adding the estimated right atrial pressure value, obtained from the vena cava collapsibility index (Doppler and M-Mode modality);
- For EDT, RV diameter, RV wall thickness, and PASP parameters, normal vs. altered classes were generated.

### 2.3. Statistical Analysis

Data were summarized as mean value with standard deviation or frequencies (number) and evaluated for Gaussian distribution (Shapiro–Wilk test) before applying statistical analysis using the Graph Pad Software (Prism 4, San Diego, CA, USA) and the R programming language (Vienna, Austria, 2018) [29]. Depending on this result, parametric (Student’s *t* test) or non-parametric (Wilcoxon test) tests were applied for data comparison between two groups. For comparisons between different groups in terms of relative frequencies, we used the Pearson chi-squared test applying the Monte Carlo correction in the case of low numbers. ANOVA was used for comparisons of three groups or more. If significant, the Holm–Bonferroni posthoc method was used to confirm differences between groups. The risk of EDT alterations was evaluated by odd ratio (OR) between oxygen desaturators vs. non oxygen desaturators and considering medium/severe Mdes vs. mild Mdes. For all analyses, *p* < 0.05 was considered statistically significant.

## 3. Results

Of the 2153 patients assessed by nCRP at our facility between 2007 and 2016, 1774 resulted as confirmed OSA, of which 128 were without comorbidities and eligible for the study. A further 25 patients were successively excluded due to technical ETT reasons. Hence, statistical analysis was performed on 103 patients (Figure 1). 

Table 1 shows patient characteristics, nCRP and ETT parameters (mean and standard deviations), while Table 2 shows the subdivisions into classes (based on sex, age, ETT, and nCRP parameters) with relevant discriminating values of classes and absolute frequencies.

### 3.1. Primary Aim

**Figure 2:**
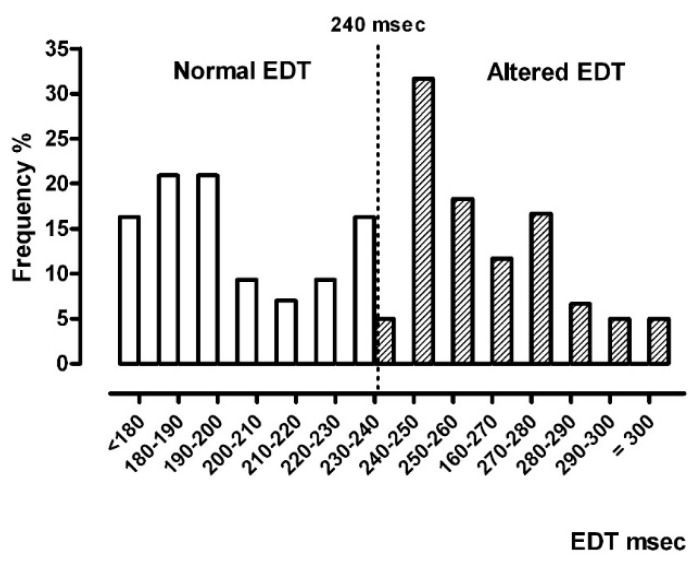
Frequency distribution of EDT values in the EDT classes: normal EDT (white) and altered EDT (shadow). Legend: EDT = trans-tricuspid E-wave deceleration time.

The diastolic RV function as evaluated by EDT was altered (EDT ≥ 242 ms) in 58.3% of patients. The altered patients showed mild RVD dysfunction (EDT median 256 ms; interquartile range, IQR 247–264) with respect to the preserved RVD function group (EDT median 192 ms; IQR 183–221). Figure 2 shows frequency distributions of normal vs. altered EDT.

Analyzing the association between altered EDT and demographic/minor comorbidities data, we found:

- No association with sex (*p* = 0.847) or presence of systemic hypertension (*p* = 1.000);
- No significant difference in age: 61.9 years in normal EDT vs. 62.1 years in altered EDT (*p* = 0.948);
- A significant difference in body mass index (BMI): 30.9 in normal EDT vs. 34.6 Kg/m^2^ in altered EDT (*p* = 0.013; 95%CI −4.90:−0.70).

### 3.2. Secondary Aims

No significant differences were found between altered and normal EDT classes in relation to AHI, ODI, and apnea duration (Table 3). No significant association between altered EDT function and AHI-OSA severity classes (*p* = 0.683) was found either. However, altered EDT patients differed significantly from normal EDT patients concerning the mean SpO_2_, T90, and Mdes parameters (Table 3).

**Figure 3:**
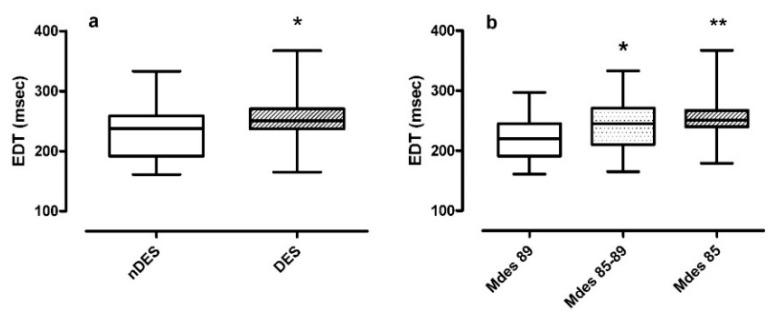
EDT values boxplot stratified for T90 classification (DES/nDES) (**a**) and for Mdes severity (mild/moderate/severe) (**b**). Legend: EDT= trans-tricuspid E-wave deceleration time; DES = oxygen desaturators; nDES= non oxygen desaturators; Mdes= mean desaturation peak values of nocturnal events; Mdes 89 = Mdes ≥ 89% = mild group; Mdes 85–89= Mdes < 89% and >85%= moderate group; Mdes 85 = Mdes ≤85% = severe group; DES vs. nDES: * *p* < 0.05; Mdes 85–89 vs. Mdes 89; * *p* < 0.05 and Mdes 85 vs. Mdes 89: ** *p* < 0.01.

Moreover, EDT values were significantly different in classes according to time spent with desaturation (T90) and Mdes:

- Non-desaturators vs. desaturators in Figure 3 panel a. The median EDT in desaturators was 250.0 ms compared to the median EDT in non-desaturators 238.3 ms (*p* = 0.027);
- Mdes severity classes in Figure 3 panel b. The median EDT value increased as Mdes decreased, from 219.5 ms in mild to 245.0 ms in moderate and reaching 251.0 ms in severe Mdes (*p* = 0.005). Post-hoc analysis confirmed a significance between moderate and mild Mdes (*p* = 0.032) and severe and mild Mdes (*p* = 0.001).

**Figure 4:**
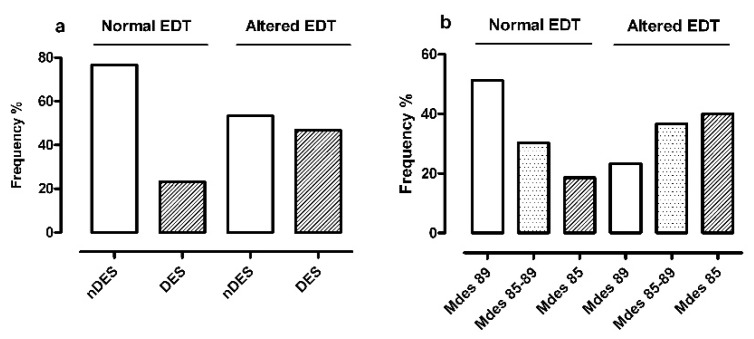
Frequency distribution of nDES/DES classes(**a**) and of Mdes severity classes (**b**) in relation to normal/altered EDT. Legend: EDT = trans-tricuspid E-wave deceleration time; DES = oxygen desaturators; nDES = non oxygen desaturators; Mdes = mean desaturation peak values of nocturnal events; Mdes 89 = Mdes ≥ 89% = mild group; Mdes 85–89 = Mdes < 89% and >85% = moderate group; Mdes 85 = Mdes ≤85% = severe group.

Concerning the associations between EDT classes and classes derived from nCRP parameters, significant results were found in regards to time spent in desaturation (T90) and the severity classes of Mdes:

- Non-desaturators and desaturators (Figure 4 panel a) resulted differently distributed in the two EDT classes (*p* = 0.026): there was a lower frequency of desaturators in normal EDT (10/43 cases, 23%) than altered EDT (28/60 cases, 47%). Of note, the OR for altered EDT in desaturators with respect to non-desaturators was 2.86 (95%CI 1.12:7.72);
- The severity classes of Mdes (Figure 4 panel b) were characterized by an exactly opposite distribution in the two EDT classes (*p* = 0.008): severe group (Mdes 85) was the most represented in altered EDT (24/60 cases, 40%) and the least represented in normal EDT (8/43 cases, 19%). In addition, the OR for altered EDT in medium/severe Mdes with respect to mild Mdes was 3.40 (*p* = 0.007; 95%CI 1.36:8.77).

We found that RV wall thickness was significantly greater in desaturators than non-desaturators (*p* = 0.008; 95%CI −2.35 × 10^−5^:−3.12 × 10^−5^), while it did not significantly differ in the AHI and Mdes severity classes (*p* = 0.622 and *p* = 0.206, respectively).

The RV systolic function evaluated with TDI resulted (RV-Sm in Table 1) in the normal range (peak systolic velocity of tricuspid annulus > 0.09 m/s).

The estimated values of diurnal PASP were in most cases (82.6%) normal, and no significant differences between the two PASP classes (normal vs. pathological) were found regarding EDT (*p* = 0.520) and the nCRP parameters. 

### 3.3. Influence of Minor Comorbidities

The presence of systemic arterial hypertension (77.7% of cases) did not lead to significant differences in EDT values (EDT hypertension yes vs. no; *p* = 0.440) or nCRP parameters. It should be noted that patients with hypertension were all on medical therapy and with controlled blood pressure values. However, there was an association between systemic hypertension and severity of Mdes (*p* = 0.037). In particular, the percentage of cases classified as mild (Mdes ≥ 89%) were 56.5% in patients without hypertension and 28.7% in patients with hypertension, while cases classified as moderate (Mdes 85–89%) were 17.4% and 38.7%, respectively.

## 4. Discussion

We have shown that the RVD function, evaluated by EDT, was altered in 58.3% of OSA patients and this correlated with the degree of nocturnal hypoxemia severity. With respect to patient characteristics and comorbidities, only an association with higher BMI was found. In particular, EDT alteration was related to hypoxia, both in terms of the severity of the oxyhemoglobin desaturation peaks (Mdes) and of the amount of time spent in desaturation (“desaturators” vs. “non-desaturators”).

The pathophysiological mechanisms hypothesized to explain the association between OSA and cardiovascular pathologies are multiple and probably interconnected. It is well known that, during apnea events and intermittent hypoxemia, a series of physiological effects occurs at the same time. These include exaggerated negative intrathoracic pressure, sympathetic activation with a consequent increase in cardiac work, an enhanced venous return and increase in oxygen requirements with potential ischemia and oxidative stress, as well as endothelial dysfunction, metabolic dysregulation, and inflammation [9,30,31]. Hypoxia is a determining factor in pulmonary vasoconstriction with acute increase in RV post-load, possible long-term development of parietal hypertrophy, and systolic and/or diastolic dysfunction. 

Several studies have evaluated the cardiac function in OSA patients and shown the association between the syndrome severity and the impairment of a LV systo-diastolic performance in patients with normal daytime pulmonary pressure and preserved ejection fraction [17,18,30,31,32,33,34,35].

A few echocardiographic studies [17], but with a low number of patients, in recent years have shown that mild alterations in RV morphology and function are frequently present in OSA, even in the absence of chronic and significant pressure overload of the cardiac chamber. In severe OSA, the respiratory disorder is associated with hypertrophy and decreased RV systolic function and altered diastolic performance [9,16,17,21,36]. In particular, a variable association between OSA and diastolic dysfunction of the RV has been found [17,21,37], mostly investigated by markers different from EDT.

EDT was chosen in this study as the main marker characterizing an impaired RV relaxation, which is affected by active myocardial release capacity (process energy-dependent), by passive, intrinsic, myocardial properties, and finally by extrinsic components.

Recent studies have documented subclinical systolic dysfunction to be detectable by speckle tracking [36], real-time three-dimensional echocardiography [38], and TDI [13]. On the contrary, our results show that the identified systolic function parameter (RV-Sm), which well correlates with alternative measures of global RV systolic function, was not found significantly altered. We hypothesize that the altered RVD function could take place before the structural alteration of the myocardial wall, which is potentially connected to a detectable systolic dysfunction.

Of note, the prolonged EDT did not present significant correlations with the number of apneic events and their duration, but with the mean night saturation, desaturation duration, and with the severity of the desaturation associated peaks: These results, in accordance with Chung et al. [39], suggest a possible interaction between time spent with a low level of oxygen and risk of developing RV function alteration.

The right ventricle wall thickness in our patients was in the upper limit of the normal range and appeared to be significantly correlated with OSA severity in terms of T90 values (desaturation size) but not in terms of AHI, as found in Zakhama et al.’s study [40]. 

The estimated values of diurnal pulmonary artery pressure did not show any significant association with EDT and nCRP parameters, in line with the literature [40]; the values were mainly within the standard range. 

Finally, we found the EDT alteration to be independent of the presence of systemic hypertension, in agreement with Tugcu 2009 [37]. We observed a relation between systemic arterial pressure and the severity of the desaturation peaks: among the subjects with systemic hypertension, the most represented class was moderate Mdes (85–89%), while in the absence of hypertension more than a half of the patients were in the mild severity class (Mdes ≥ 89%). 

### 4.1. Limitations

The retrospective study design, the availability of ETT evaluations for OSA confirmed patients, the suitable imaging for retrospective EDT measure, and the consequent low number of patients, may be limitations.

### 4.2. Practical Implications

In this study we have (1) identified a non-invasive, low-cost, accurate, easy-to-acquire instrumental cardiological marker that can, in a clinical context, screen for the existence of a respiratory pathology and prompt an appropriate diagnostic–therapeutic pathway; (2) highlighted the importance of recognition and early treatment of OSA, with the aim to prevent or treat cardiovascular complications.

Further studies are needed to (1) investigate the pathophysiological substrate and cause–effect mechanism between nocturnal desaturation and cardiac modifications; (2) explore if EDT may be an early predictor of OSA severity, with subsequent implications for prevention and treatment strategies; (3) verify if the use of CPAP and NIMV could be of lasting benefit to OSA patients, not only from a respiratory but also cardiovascular point of view.

## 5. Conclusions

We have shown that, in a population of untreated ‘pure’ OSA patients, RV diastolic function, evaluated by EDT, was altered in almost 60% of cases, and it correlated with the degree of nocturnal hypoxemia severity independently of sex, age, and presence of systemic arterial hypertension but in association with high BMI. Moreover, in our selected population, significant alterations of RV systolic function were not detected and the estimated values of diurnal pulmonary artery pressure were, in most cases, normal, suggesting that RVD alteration could be a subclinical condition meriting further investigation.

## Figures and Tables

**Table 1:** Patient characteristics, nCRP, and ETT evaluations at admission.

	Mean (SD)
**Age**, years	62.04 (10.99)
**BMI**, Kg/m^2^	33.86 (6.08)
**EDT**, ms	237.03 (39.01)
**RV diameter**, mm	30.53 (4.25)
**RV wall tickness**, mm	4.46 (10.6)
**RV-Sm**, m/s	0.15 (0.03)
**PASP**, mmHg	28.65 (6.54)
**AHI**, events/h	37.51 (26.77)
**mean SpO_2_**, %	90.34 (5.10)
**T90 (TimeSpO_2_)**, %	29.10 (30.65)
**Mdes**, %	85.77 (6.24)
**Apnea duration**, s	20.42 (5.07)

Legend: nCRP = nocturnal eight-channel cardiorespiratory polygraphy; ETT = comprehensive echocardiographic examination; BMI, body mass index; EDT = trans-tricuspid E-wave deceleration time; RV = right ventricle; RV-Sm = peak systolic velocity of tricuspid anulus; PASP = pulmonary artery systolic pressure; AHI = apnea/hypopnea index; mean SpO_2_ = nighttime oxyhemoglobin saturation mean value; T90 = time elapsed with SpO_2_ value less than 90%; Mdes = mean desaturation peak values of nocturnal events, SD, standard deviation.

**Table 2:** Stratification of patient characteristics, nCRP, and ETT evaluations based on reference values or classification groups.

	Frequency (*n*.)
**Sex**, M/F	72/31
**Hypertension**, no/yes	23/80
**EDT**, normal (<240 ms)/altered	43/60
**RV diameter**, normal (<35 mm)/altered	91/12
**RV wall tickness**, normal (<5 mm)/altered	76/27
**PASP**, normal (<35 mmHg)/altered	85/18
**OSA severity** mild/moderate/severe	29/21/53
**Respiratory insufficiency**, nDES (T90 < 30%)/DES	65/38
**Mdes severity**, mild/moderate/severe	36/35/32

Legend: M/F = Male/Female; nCRP = nocturnal eight-channel cardiorespiratory polygraphy; ETT = comprehensive echocardiographic examination; EDT = trans-tricuspid E-wave deceleration time; RV = right ventricle; PASP = pulmonary artery systolic pressure; nDES = non oxygen desaturators; DES = oxygen desaturators; T90 = time elapsed with SpO_2_ value less than 90%; Mdes = mean desaturation peak values of nocturnal events.

**Table 3:** Median of nCRP parameters according to EDT classification (altered vs. normal).

	Normal EDT (*n* = 43)	Altered EDT (*n* = 60)	*p* (95%CI)
AHI, events/h	26.80	35.00	0.062 (−18.70:0.40)
ODI, events/h	28.90	37.80	0.068 (−19.70:0.40)
mean SpO_2_, %	92.50	91.25	**0.002 (0.70:3.20)**
T90 (TimeSpO_2_), %	9.10	28.45	**0.003 (−24.80:−2.90)**
Mdes, %	89.10	87.05	**0.002 (0.90:3.90)**
Apnea duration, s	19.30	19.85	0.383 (−2.50:1.00)

Legend: nCRP = nocturnal eight-channel cardiorespiratory polygraphy; EDT = trans-tricuspid E-wave deceleration time; 95%CI = 95% confidence interval; AHI = apnea/hypopnea index; ODI = oxygen desaturation index; mean SpO_2_ = nighttime oxyhemoglobin saturation mean value; T90 = time elapsed with SpO_2_ value less than 90%; Mdes = mean desaturation peak values of nocturnal events. Bold indicated *p* < 0.05.
